# The prognosis of bladder cancer is affected by fatty acid metabolism, inflammation, and hypoxia

**DOI:** 10.3389/fonc.2022.916850

**Published:** 2022-11-21

**Authors:** Yu Xiao, Junfeng Yang, Maolin Yang, Jinjun Len, Yanhong Yu

**Affiliations:** ^1^ The Affiliated Hospital, Kunming University of Science and Technology, Kunming, China; ^2^ Department of Urology, The First People’s Hospital of Yunnan Province, Kunming, Yunnan, China

**Keywords:** bladder cancer, fatty acid metabolism, inflammation, hypoxia, gene signature, immunotherapy response

## Abstract

**Background:**

The prognosis of bladder cancer (BC) is poor, and there is no effective personalized management method for BC patients at present. Developing an accurate model is helpful to make treatment plan and prognosis analysis for BC patients. Endogenous fatty acid metabolism causes cancer cells to become hypoxic, and the coexistence of hypoxia and inflammation is often characteristic of cancer. All three together influence the tumor immune microenvironment, treatment, and prognosis of BC.

**Methods:**

We used The Cancer Genome Atlas-Bladder Urothelial Carcinoma (TCGA-BLAC) cohorts as a train group to build a risk model based on fatty acid metabolism, hypoxia and inflammation-related gene signatures and performed external validation with GSE13507, GSE31684, and GSE39281 cohorts. We validated the model to correlate with the clinicopathological characteristics of patients, created an accuracy nomogram, and explored the differences in immune microenvironment and enrichment pathways.

**Results:**

We found significant differences in overall survival and progression-free survival between high- and low-risk groups, and patients in the low-risk group had a better prognosis than those in the high-risk group. In the train group, the AUCs for predicting overall survival at 1, 3, and 5 years were 0.745, 0.712, and 0.729, respectively. The 1-, 3-, and 5-year overall survival AUCs were 0.589, 0.672, and 0.666 in the external validation group, respectively. The risk score independently predicted the prognosis of BC patients with AUCs of 0.729. In addition, there was a significant correlation between risk scores and BC clinicopathological features and, in the GSE13507 cohort, we observed that BC progression and deeper invasion were associated with higher risk scores. Risk scores were highly correlated with coproptosis, pyroptosis, m7G, immune checkpoint-related genes, and immune microenvironment. In addition, we found that patients in the low-risk group responded better to immunotherapy, whereas patients in the high-risk group were more sensitive to commonly used chemotherapy drugs.

**Conclusion:**

Our findings provide new treatment decisions for BC, and can effectively predict the prognosis of BC patients, which is helpful for the management of BC patients.

## Introduction

BC is one of the most common cancers in the world, and currently ranks 10th in incidence worldwide (1). It is a disease that cannot be ignored. Radical cystectomy with lymph node dissection is currently an effective treatment for high-risk BC patients (2). With the in-depth understanding of immunotherapy, a number of cancer has achieved effective results in immunotherapy (3, 4). The application of immunotherapy in BC has gradually changed the existing management mode (5). However, the response of immunotherapy for BC is not so satisfactory, and some patients with BC may not benefit from it (6), so personalized management is very critical. Tumor microenvironment (TME) is heterogeneous and is a complex ecosystem, which is considered to be one of the main markers of epithelial cancer and is related to the occurrence, development, and drug resistance of tumors (7). The occurrence of drug resistance may be related to the lack of drug-sensitive immune cells in the TME.

Cell proliferation is one of the common features of all cancers, and limiting fatty acid synthesis membranes and signaling molecules can control cancer cell proliferation (8). Fatty acid metabolism in the TME affects tumor immunotherapy response and prognosis through regulation of immune cells and hypoxia of cancer cells (9, 10). Hypoxia is common in malignant tumors. Hypoxia plays an important role in chronic tumor inflammation, affecting the TME and eventually transforms neutrophils and monocytes into tumor-associated neutrophils and tumor-associated macrophages (11). A central manifestation of malignancy is metabolic dysfunction, chronic inflammation plays an important role in tumor development, and metabolic modifications of immune checkpoints and inflammation influence the therapeutic strategy of tumors (12). Fatty acid metabolism, hypoxia, and inflammation work together to affect the occurrence, development, and prognosis of tumors (**Supplementary Figure S1**).

In the past few years, there has been no significant progress in the treatment strategy of BC, and there is no comprehensive assessment based on fatty acid metabolism, inflammation, and hypoxia. Model construction based on the three is necessary.

## Materials and methods

### Date sources

RNA- sequencing transcriptome profiling harmonized to the fragments per kilobase million (FPKM) of 411 BC samples, 19 normal samples, nucleotide variation (Masked Somatic Mutation), and corresponding clinical data were downloaded from the TCGA database (https://portal.gdc.cancer.gov/repository). The GSE13507, GSE31684, and GSE39281 cohort that contained 352 BC samples were downloaded from Gene Expression Omnibus (GEO) (https://www.ncbi.nlm.nih.gov/geo/). Fatty acid metabolism, inflammation, and hypoxia-related genes (*n* = 309, 200 and 200, respectively) were retrieved from previous studies (13–15). For TCGA-BLAC cohort, FPKM values were transformed into transcripts per million (TPM) (16). Combining the GSE13507, GSE31684, and GSE39281 cohort, we remove normal samples and zero survival time samples and use the “sva” package of R software for batch correction. All patients’ clinical information is described in (**Supplementary Table S1**).

### Extraction of differentially expressed genes

The expression levels of fatty acid metabolism, inflammation, and hypoxia-related genes were extracted from the TCGA-BLAC cohort. Differentially expressed genes (DEGs) between BC and normal bladder tissue were identified using the Wilcoxon test according to LogFC > 1 and FDR < 0.05 would be considered as DEGs. LogFC > 0 indicated upregulated genes, otherwise, downregulated genes. In addition, we visualized the above DEGs with volcano and heat map, respectively. The obtained DEGs were intersected with GSE13507, GSE31684, GSE39281, and TCGA-BLCA cohort (*n* = 195); the Venn diagram was drawn. We used the “clusterprofiler” package of R software for enrichment analysis of Gene Ontology (GO), functional annotation, and Kyoto Encyclopedia of Genes and Genomes (KEGG).

### Construction and verification of the risk model

The expression levels of the intersection genes were extracted and univariate Cox regression analysis was performed to determine the factors associated with BC overall survival. We use the TCGA cohort as the train group and the GEO cohort as the external validation group, and the subsequent risk model was constructed using the train group. The risk of overfitting was minimized using Lasso Cox regression with 10-fold cross-validation analysis using the “glmnet” R package (17).

Risk Score =Σ(Expi * Coefi)

Expi and Coefi represent the expression level and risk coefficient of each gene, respectively. According to the median risk score of train group, the samples were divided into high- and low-risk groups and subsequent survival analysis was carried out. Depicting the ROC curve and calculate the area under the curves (AUCs) for 1, 3, and 5 years with the “timeROC” package of R software. Principal components analysis (PCA) was carried out with the “ggplot2” package of R software. An interaction network for high- and low-risk groups DEGs was generated by the STRING database (https://cn.string-db.org/). GO and KEGG gene symbols were obtained from Gene Set Enrichment Analysis (GSEA) database (http://www.gsea-msigdb.org/gsea/downloads.jsp), and Gene Set Variation Analysis (GSVA) enrichment analysis of high and low risk groups was carried out by using “limma” R package.

### Correlation between risk score and BC patients clinicopathological features

We performed a stratified analysis according to age, sex, TNM stage, grade, and stage of BC patients to determine the objective predictive power of risk score, and we analyzed the correlation between risk score and BC progression.

### Establishment and verification of the nomogram

Based on the BC patient’s clinicopathological characteristics and risk score, an accurate nomogram was built using the “RMS” R package, improving the utility of risk score. Each variable in the figure could be scored individually, and all scores were added to obtain a total score to predict the survival of BC patients.

### Correlation between risk score and immune cells

We assessed the correlation between risk scores and 22 types of immune cells and observed differences in immune function between the two groups. The ESTIMATE algorithm was used to calculate the stromal score, immune score, and ESTIMATE score of all BC samples between high- and low-risk groups.

### Mutation and cancer stem cell analysis

We used the “maftools” R package to evaluate somatic mutations in high and low-risk groups (18). We also assessed the prediction of tumor mutational burden (TMB) in BC patients overall survival.

### Correlation between risk score and coproptosis, pyroptosis, m7G, and immune checkpoint–related genes

We analyzed the expression differences of coproptosis, m7G, pyroptosis, and immune checkpoint–related genes in high and low-risk groups to further verify the validity of the risk model.

### Predicting chemotherapeutic drug sensitivity and immunotherapy response

We evaluated the response of risk score to commonly used chemotherapeutic drugs for BC; the half-maximal inhibitory concentration (IC50) was first calculated in all BC patients, using the “pRRophetic” R package. The Wilcoxon rank test was then used to compare the difference in IC50 between the low and high-risk groups. We also observed the differential expression of microsatellite instability (MSI) in high- and low-risk groups. The immunotherapy score was downloaded from the The Cancer Immunome Atlas database (https://www.tcia.at/patients), and then we evaluated the differences in immunotherapy between high and low-risk groups.

### Statistical analysis

All the statistical analyses and picture drawing are done by R software (version 4.1.2). *P* < 0.05 is considered to have the statistical difference, the Benjamini–Hochberg (BH) multiple test correction is used to calculate the adjusted *P*- value.

## Results

### Differentially expressed fatty acid metabolism, hypoxia, and inflammation-related genes

**Figure 1:**
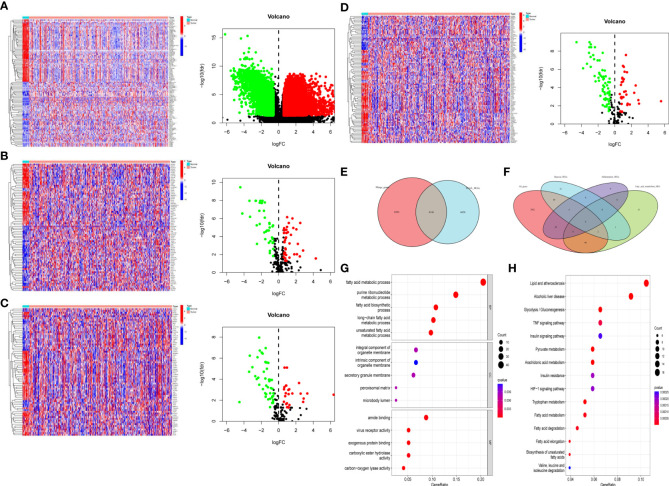
Differentially expressed fatty acid metabolism, hypoxia, and inflammation-related genes. **(A)** All the BC DEGs in the TCGA-BLAC cohort **(B)** The fatty acid metabolism-related DEGs in the TCGA-BLAC cohort. **(C)** The inflammation-related DEGs in the TCGA-BLAC cohort. **(D)** The hypoxia-related DEGs in the TCGA-BLAC cohort. **(E)** Venn diagram of the intersection genes among the TCGA-BLBA DEGs and merge genes. **(F)** Venn diagram of the intersected fatty acid metabolism, hypoxia, and inflammation-related DEGs. **(G)** GO enrichment based of integration DEGs. **(H)** KEGG enrichment based of integration DEGs.

First, we extracted all the DEGs in the TCGA cohort, and then we extracted the DEGs of fatty acid metabolism, inflammation and hypoxia (*n* = 78, 72, and 97), and drew volcano maps and heat maps (**Figures 1A–D**), respectively. Finally, we got 4,228, downregulated DEGs, among which fatty acid metabolism, hypoxia and inflammation-related DEGs were *n* = 38, 64, and 47, 6,544 upregulated DEGs, among which fatty acid metabolism, hypoxia and inflammation-related DEGs were *n* = 40, 33, and 25, respectively. We extracted the intersection genes of TCGA DEGs and external validation cohort genes (**Figure 1E**) and took the intersection with the fatty acid metabolism, hypoxia, and inflammation-related DEGs (**Figure 1F**). We performed GO and KEGG enrichment analysis on 195 differentially co-expressed genes (**Supplementary Figures S2A, B**); GO enrichment analysis showed that the most significant change were fatty acid metabolic process and purine ribonucleotide metabolic process (**Figure 1G**). KEGG enrichment analyses showed that the most significant change was lipid and atherosclerosis and alcoholic liver disease (**Figure 1H**).

### Construction and validation of the risk score

**Figure 2:**
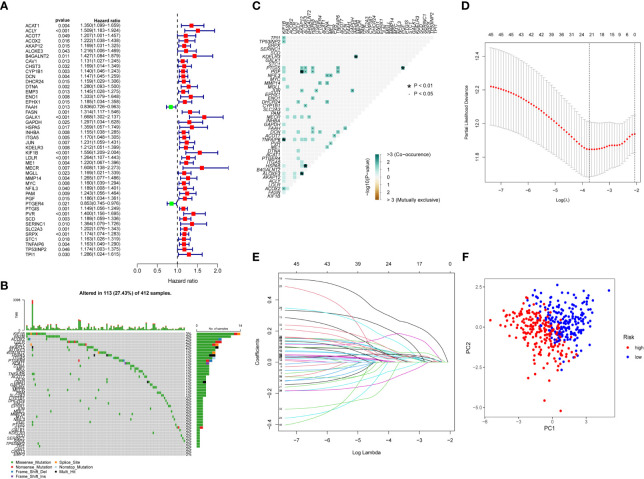
Construction of risk score. **(A)** Univariate Cox regression analysis forest plot. **(B)** Somatic mutation of prognosis-related genes in BC patients, with colors representing different mutation types. **(C)** Co-mutation correlation among prognosis-related genes. **(D, E)** Twenty-two prognosis-related genes were retained by LASSO regression analysis. **(F)** PCA analysis of high- and low-risk group patients.

We performed univariate Cox regression analysis to find the BC prognosis-related gene and draw a forest plot (**Figure 2A**). The waterfall map shows that the somatic mutation rate of prognosis-related genes in the TCGA-BLAC cohort is 27.43% (**Figure 2B**), and some genes have a co-mutation relationship with each other (**Figure 2C**). LASSO regression analysis further optimized our selection of risk model genes and, finally, 22 genes were included in the risk model (**Figures 2D, E**). Among them, 19 high-risk genes and three low-risk genes were obtained (**Supplementary Table S2**), Risk Score =Σ(Expi * Coefi), and we plotted a risk heatmap in the train group and external validation group, with the increase of risk score, the number of deaths gradually increased in the train group and external validation group (**Supplementary Figures 3A–F**). PCA showed that there were significant differences between high- and low-risk groups (**Figure 2F**).

### Prognostic analysis of risk score

**Figure 3:**
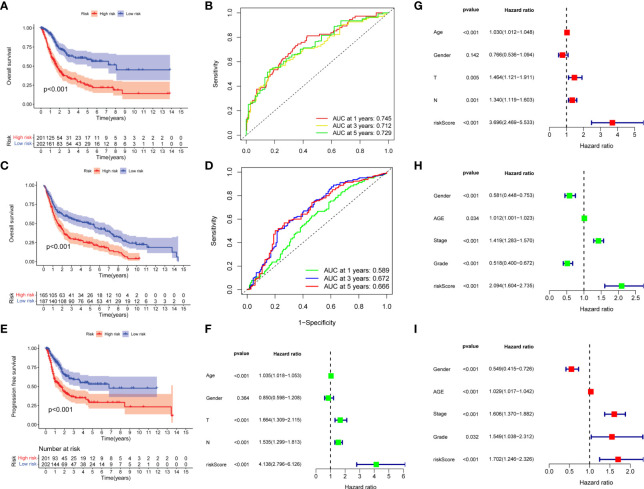
Prognostic analysis of risk score. **(A, B)** Overall survival curve and ROC curve for train group. **(C, D)** Overall survival curve and ROC curve for external validation group. **(E)** Progression- free survival curve for train group. **(F, G)** Univariate and multivariate Cox regression analysis of risk score for predicting overall survival in the train group. **(H, I)** Univariate and multivariate Cox regression analysis of risk score for predicting overall survival in the external validation group.

Kaplan–Meier survival curve showed that overall survival, the AUCs of 1-, 3-, and 5-year overall survival was 0.745, 0.712, and 0.729, respectively (**Figures 3A, B**). In addition, the same results were obtained in the external validation group, the AUCs of 1, 3, and 5-year overall survival was 0.589, 0.666, and 0.672, respectively (**Figures 3C, D**). The progression- free survival in the low-risk group was better than those in the high-risk group (**Figure 3E**). Univariate and multivariate Cox regression analyses showed that risk scores were good predictors of overall survival in BC patients (**Figures 3F, G**), and we performed external validation (**Figures 3H, I**).

### Clinicopathological correlation analysis of risk score

**Figure 4:**
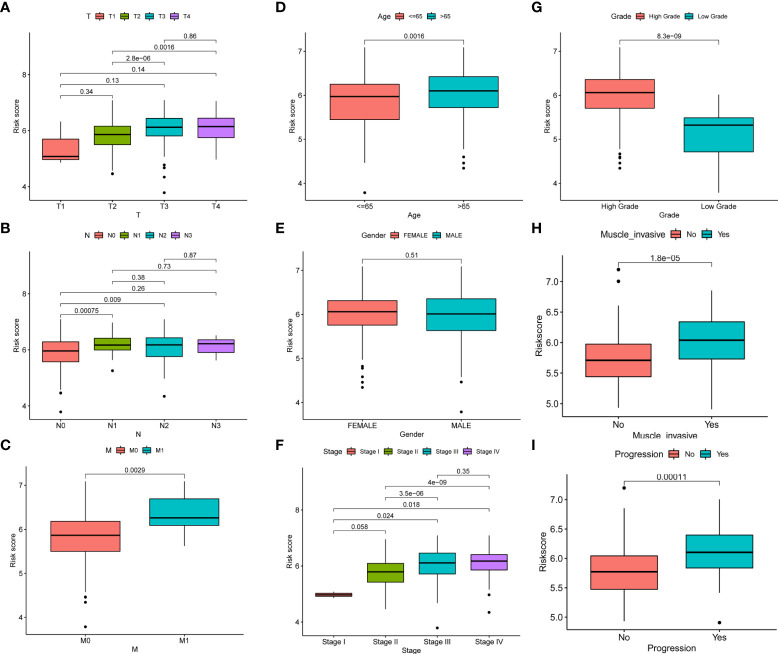
Correlation between risk score and clinicopathological features of BC. **(A–I)** Correlation between risk score and TNM stages, age, gender, stage, grades, depth of tumor invasion, and tumor progression of BC patients.

In order to better apply the risk score to the clinic, we analyzed the correlation between the risk score and the clinicopathological features of BC. The results showed that there was a significant correlation between TNM stages, age, and risk score, but risk score was not correlated with gender (**Figures 4A–E**). In addition, we observed higher stage, grades, depth of tumor invasion, and tumor progression in high-risk patients (**Figures 4F–I**). It also showed that the high-risk group had a worse prognosis.

### Construction and validation of a nomogram

**Figure 5:**
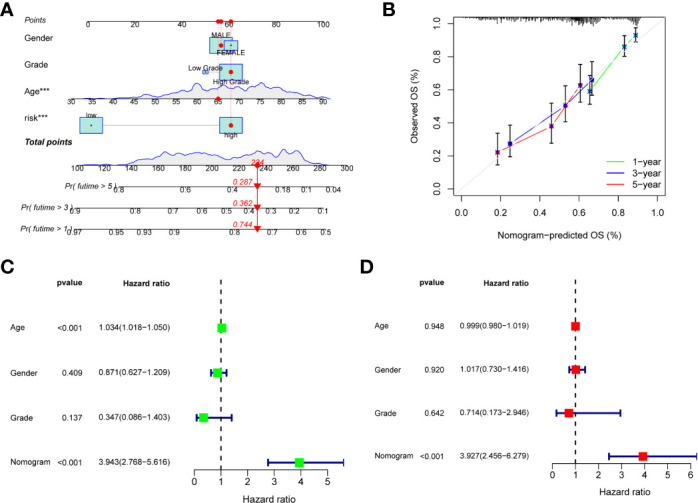
Construction of a nomogram. **(A)** Nomogram for predicting the 1-, 3-, and 5-year overall survival in the train group, the red dots represent the clinicopathological features of the sample patient. **(B)** Calibration curves of the nomogram for train group. **(C, D)** Univariate and multivariate Cox regression analysis of nomogram for predicting overall survival in the train group.

To better apply the risk score, we constructed an accurate nomogram based on sex, age, grade, and risk score to predict 1-, 3-, and 5-year overall survival in BC patients (**Figure 5A**). In addition, the calibration plots showed that our constructed nomogram had good predictive performance (**Figure 5B**). Univariate and multivariate Cox regression analysis showed that risk scores were good predictors of overall survival in BC patients (**Figures 5C, D**). In the external validation group, nomogram remained reliable in predicting overall survival (**Supplementary Figures S4A–C**).

### Correlation between risk score and BC immune microenvironment

**Figure 6:**
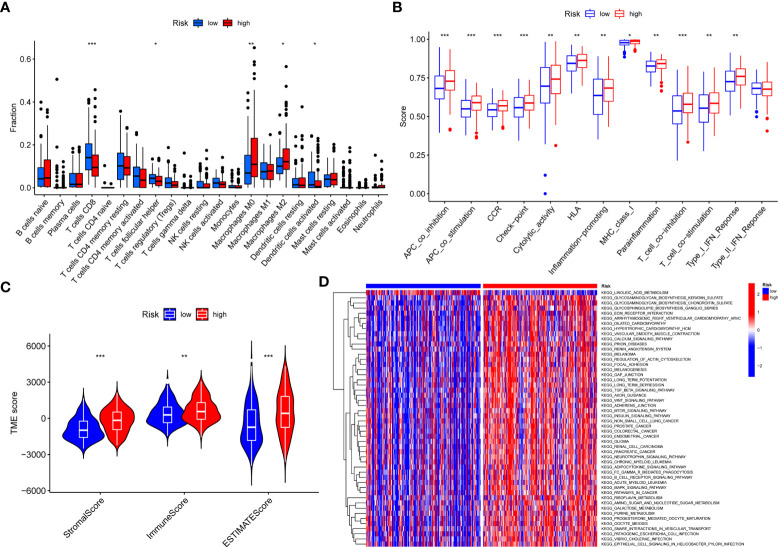
The correlations between risk score and TME. **(A)** Correlations between risk score and immune cells types. **(B)** Correlations between risk score and immune cell function. **(C)** Differences in TME scores between high and low-risk groups. **(D)** GSVA enrichment analysis for risk score. *P < 0.05, **P < 0.01, ***P < 0.001.

We found significant differences in expression between high- and low-risk groups in T- cell CD8, T- cell follicular helper, Macrophages M0, Macrophages M2, and dendritic cells activated, with T- cell CD8, T- cell follicular helper and dendritic cells activated being highly expressed in the low-risk group and Macrophages M0 and Macrophages M2 being highly expressed in the high-risk group (**Figure 6A**). In addition, immune function was more active in the high-risk group (**Figure 6B**). Furthermore, there were significant differences in TME between high- and low-risk groups. We found that StromalScore, ImmuneScore, and ESTIMATEScore were highly expressed in high-risk group (**Figure 6C**). GSVA enrichment analysis showed that most of the enrichment pathways were highly expressed in high-risk group (**Figure 6D**).

### Mutation analysis

**Figure 7:**
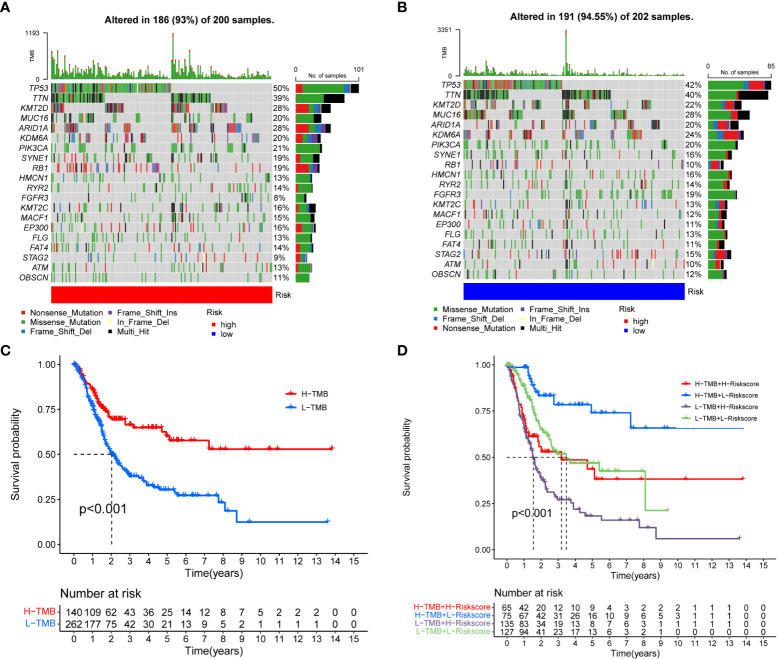
Correlation between risk score and TMB. **(A, B)** Somatic mutation features of BC patients in high- and low-risk groups, with colors representing different mutation types. **(C, D)** Survival difference of patients with different TMB.

Accumulative evidence showed that high TMB can be used as a marker of cancer immunotherapy, affecting immunotherapy sensitivity and patient prognosis (19, 20). The waterfall chart showed somatic mutations in the high- and low-risk groups (**Figures 7A, B**). Both groups had significant mutations, and the TP53 mutation rate was the highest, 50 and 42%, respectively. The TMB in the low-risk group was higher than that in the high-risk group. We observed that patients with high TMB had significantly better prognosis than patients with low TMB (**Figure 7C**). When TMB and risk score were combined, the prognosis was significantly best in the high TMB + low- risk group (**Figure 7D**). Wu et al. showed that tumor patients with high TMB had better overall survival and progression free survival and were more likely to benefit from immunotherapy. These evidences are consistent with our results.

### Expression of coproptosis, pyroptosis, m7G, and immune checkpoint-related genes

**Figure 8:**
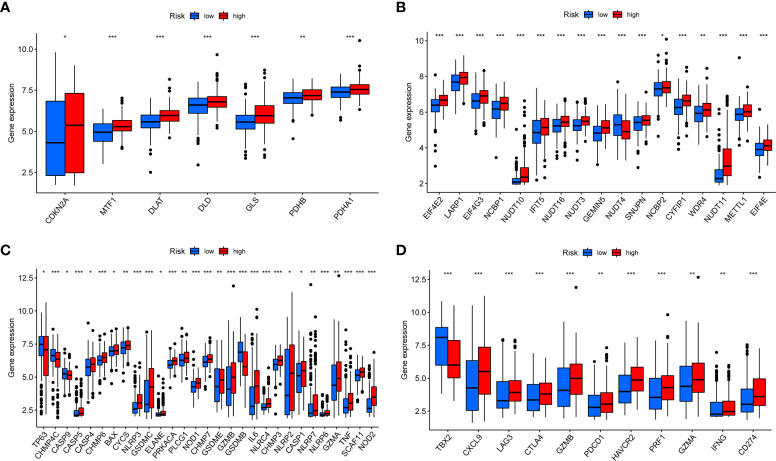
Expression of coproptosis, m7G, pyroptosis, and immune checkpoint-related genes in risk score **(A–D)**. Expression differences of coproptosis, m7G, pyroptosis, and immune checkpoint-related genes in high- and low-risk groups. *P < 0.05, **P < 0.01, ***P < 0.001.

Previous studies had shown that m7G (21) and pyroptosis (22) were closely related to the occurrence and the development of tumors. Coproptosis is a newly discovered mechanism of cell death, which depends on the full binding of lipoylated components in the tricarboxylic acid cycle to copper (23). We observed that most coproptosis, m7G, and pyroptosis-related genes were significantly different in the high- and low-risk groups, and most genes were highly expressed in the high- risk group (**Figures 8A–C**), which also corresponds to the previous conclusion. With the successful application of immunotherapy in many kinds of cancer, immunotherapy had gradually become an indispensable method for the treatment of cancer. In the risk score, we found that there were significant differences in the expression of PD-1, PD-L1, and CTLA4 between high- and low-risk group, and most of the immune checkpoint-related genes were highly expressed in the high-risk group (**Figure 8D**).

### Functional analysis

**Figure 9:**
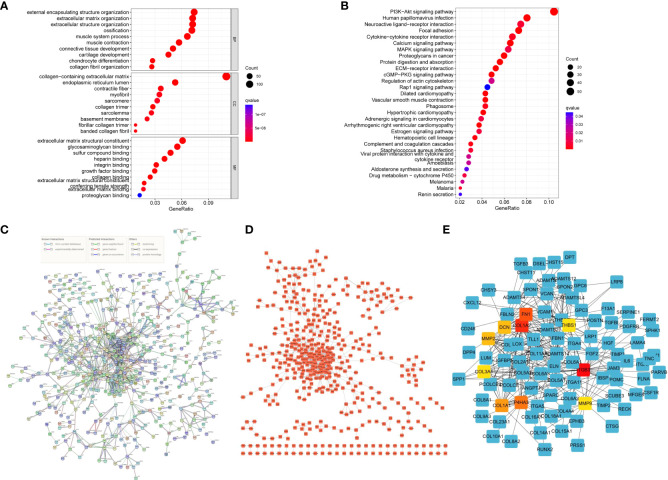
Difference analysis between high- and low-risk groups. **(A, B)** GO and KEGG enrichment analysis between high- and low-risk groups. **(C)** Interaction network of DEGs in the high- and low-risk groups. **(D)** The expression of DEGs in high- and low-risk groups, red represents upregulation. **(E)** The top 10 most significant hub genes.

Pathway differences between high- and low-risk groups showed that the most significant pathways were PI3K−Akt signaling pathway, human papillomavirus infection, and neuroactive ligand−receptor interaction (**Figures 9A, B**). Second, we analyzed the interaction network of DEGs in the two group patients (**Figure 9C**). To further search for potential prognosis- related genes of bladder cancer (BC), we visualized the interaction network with Cytoscape (v3.9.1) software and found that all node genes were upregulated genes (**Figure 9D**) and used the cytoHubba to find the top 10 hub genes (**Figure 9E**). Interestingly, we found that the expression levels of 10 hub genes were negatively correlated with overall survival in BC patients (**Supplementary Figure S5**).

### Analysis of chemotherapeutic drug sensitivity and immunotherapy response

**Figure 10:**
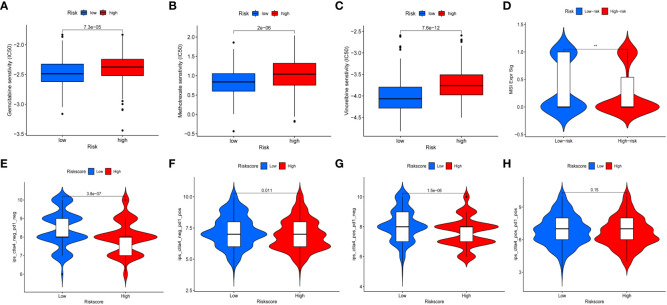
Chemotherapeutic drug sensitivity and immunotherapy response. **(A–C)** Differences in sensitivity of chemotherapy drugs in BC between high- and low-risk groups. **(D)** The expression of MSI in high- and low-risk groups. **(E–H)** Differences in the immunotherapeutic effects between the high- and low-risk groups.

We further assessed risk score for commonly used chemotherapeutic drugs sensitivity and immunotherapy response. The results showed that chemotherapeutic drugs may be more sensitive to high-risk group, such as Gemcitabine, Methotrexate, and Vinorelbine (**Figures 10A–C**). MSI is highly expressed in low-risk group (**Figure 10D**). The results of the four immunotherapy groups showed that the low-risk group may have a higher immune response (**Figures 10E–H**). In addition, we selected four genes (FASN, GALK1, MECR, and MYC) for constructing risk model for further immunohistochemistry verification (https://www.proteinatlas.org/search/), and the results showed that there were differences in gene expression between tumor and normal tissues (**Supplementary Figure 6**).

## Discussion

BC is one of the most common tumors, and the most effective treatment is still surgery and chemotherapy. Neoadjuvant chemotherapy combined with radical cystectomy is the gold standard for the treatment of muscular invasive BC (24), but about 35% of the patients may progress to metastatic BC after operation (2). There are a lot of evidences that BC is highly responsive to cisplatin-based combined chemotherapy (25, 26). Immune checkpoint inhibitors have been used in clinic, and have achieved good results in a variety of tumor treatment, but the current immunotherapy for BC is not widely used compared with other tumors (27, 28). We need new evidence to improve the current treatment.

Tumors heterogeneity led to different biological characteristics, and personalized treatment was often the key to the benefit of patients. The current risk score is relatively single, often ignoring the results of the joint action of many factors. Fatty acid metabolism, hypoxia, and inflammation often interact with each other, together affecting the occurrence, development, and prognosis of tumors; our risk score is necessary.

The poor efficacy of immunotherapy is related to the damage of immunosuppressive activity caused by metabolic reprogramming in TME; the key processes of immune cell survival and function are regulated by fatty acid metabolism, which may lead to poor prognosis (9, 29). Hypoxia can be considered as the first TME marker, which affects the metabolic process and almost all cell groups, thus affecting the proliferation and metastasis of tumor cells (30, 31). Inflammation is closely related to the tumor, and there is often an inflammatory environment around the tumor. Inflammatory cells in TME release most cytokines, which are beneficial to the survival and proliferation of tumor cells (32). Fatty acid metabolism was involved in palmitoylation, which promoted the binding of MYD88 to IRAF4 and activates NF-KB signaling, thus altering neutrophil metabolism and regulating inflammation (33). Hypoxic TME promoted tumor inflammatory response and enhanced immune tolerance (34). Changed in oxygen regulation mechanism affected tumor metabolism and accelerated tumor progression (35). In summary, the TME and biological process are also affected by fatty acid metabolism, hypoxia, and inflammation. Our research may help to improve the current treatment strategies for BC.

First, we screened out differential genes using univariate Cox regression analysis and identified 195 differentially co-expressed genes. Afterward, we identified 49 BC prognosis-related genes from differentially co-expressed genes and performed LASSO regression analysis and finally identified 22 genes for model building. Based on the median gene expression of the train group, we divided patients into high- and low-risk groups and performed external validation. There was a significant difference in survival between the two groups, and the low-risk group had a better prognosis than the high-risk group. In addition, there were differences in the immune microenvironment, chemotherapeutic drug sensitivity, and immunotherapy response between the two groups.

To understand the most significant functions and pathways of differentially co-expressed genes, we performed GO and KEGG enrichment analysis. We observed that the somatic mutation rate of prognostic-related genes in BC was 27.43%, and some genes had co-mutation relationships. There were significant differences in overall survival and progression- free survival between high- and low-risk groups, and the prognosis of patients in low-risk group was better. In the train group, the AUCs of 1, 3, and 5 years were 0.745, 0.712, and 0.729, respectively. With the increase of risk score, the number of death patients increased gradually. In order to make the risk score better applied in clinic, we constructed an accurate nomogram. Univariate and multivariate independent prognostic analysis showed that both risk score and nomogram could predict the prognosis of BC patients. The same conclusion was obtained in the external verification group. Furthermore, we found that, in addition to gender, there was a correlation between risk score and clinicopathological characteristics of patients, and a higher risk score meant a worse prognosis.

We further analyzed the correlation between the risk score and the TME. Among 22 types of immune cells, Macrophages M0 and Macrophages M2 were highly expressed in the high-risk group. Macrophages were variable with tumor progression (36). In the initial stage, macrophages were often characterized by the accumulation of Macrophages M1. With the change of TME, Macrophages M1 was gradually polarized to Macrophages M2. Macrophages M2 could directly induce the immune response of BC, which was related to the progression of BC and the inefficacy of Bacillus Calmette-Guerin therapy (37). Some studies had shown that the activation of ERK pathway could stimulate Macrophages M0 to differentiate into Macrophages M2, thus promoting tumor progression (38). In the high-risk group, the immune cell function was more active and had higher StromalScore, ImmuneScore and ESTIMATEScore. In addition, GSVA enrichment analysis showed that the high-risk group was significantly enriched in most pathways.

Somatic mutations were prevalent in the high- and low-risk groups, and the low-risk group had a higher TMB. Furthermore, we observed that a higher TMB was associated with a better prognosis. When TMB and risk score were combined, the high TMB + low- risk score group had the best prognosis. Previous evidence showed that a higher TMB may have a better prognosis (39). High TMB may be a marker of immunotherapy, and patients with high TMB may benefit more from immunotherapy (40, 41).

Copper was a cofactor for essential enzymes in organisms and played an indispensable role in the body (42). Recent evidence showed that copper-induced cell death differs from known cell death pathways and that altered copper homeostasis may contribute to disease (23). M7G and are the types of RNA modification, which affect the occurrence and development of tumors (43, 44). Pyroptosis is an inflammatory form of cell death accompanied by an immune response, and unlike coproptosis, pyroptosis is mediated by the GSDM protein family and affects tumor progression and therapy (45). Therefore, we investigated the correlation between risk score and coproptosis and m7G and pyroptosis. We found that most genes were highly expressed in the high-risk group, and the m7G-related gene METTL1 (46), which had been shown to affect the prognosis of BC, was also highly expressed in the high-risk group. These results further demonstrate the validity of the risk score.

As said before, immunotherapy provides a new solution for tumor treatment. The effectiveness of immunotherapy depends to some extent on the responsiveness of CTLA-4, PD-1, and PD-L1 (47). However, immunotherapy for BC patients is currently ineffective, and new evidence is urgently needed to guide BC immunotherapy. In this study, we observed that CTLA-4, PD-1, and PD-L1 were all highly expressed in the high-risk group. We further assessed the sensitivity of the risk score to chemotherapeutic drugs and immunotherapy, found that the high-risk group appeared to be more sensitive to chemotherapy, and the low-risk group seemed to be more suitable for immunotherapy. This echoes the previous conclusion that the high-risk group has high expression of Macrophages M0 and Macrophages M2, and the low-risk group had a higher tumor mutation burden and was more likely to benefit from immunotherapy.

Our study had some limitations, all data sources were from public databases, so we tried to ensure a sufficient sample. Second, subsequent external validation was necessary. However, it was undeniable that a comprehensive assessment of the tumor to build a model is necessary.

## Conclusions

Risk model based on fatty acid metabolism, inflammation, and hypoxia could effectively guide the treatment of BC patients. Novel risk model could predict BC prognosis.

## Data availability statement

The datasets presented in this study can be found in online repositories. The names of the repository/repositories and accession number(s) can be found in the article/**Supplementary Material**.

## Ethics statement

Ethical review and approval was not required for the study on human participants in accordance with the local legislation and institutional requirements. Written informed consent from the patients/participants or patients/participants’ legal guardian/next of kin was not required to participate in this study in accordance with the national legislation and the institutional requirements.

## Author contributions

YX, YY and JY designed the study and wrote the manuscript, YY collected relevant date and information, MY and JL analyzed date. All authors contributed to the article and approved the submitted version.

## Funding

This study were supported by Special Project of Famous Doctors of Ten Thousand Talents Plan (YNWR-MY-2020-031) and Health Internal Institutions (2018NS0255) of Yunnan Province.

## Acknowledgments

Thanks to database developers and data contributors for their contributions to this article.

## Conflict of interest

The authors declare that the research was conducted in the absence of any commercial or financial relationships that could be construed as a potential conflict of interest.

## Publisher’s note

All claims expressed in this article are solely those of the authors and do not necessarily represent those of their affiliated organizations, or those of the publisher, the editors and the reviewers. Any product that may be evaluated in this article, or claim that may be made by its manufacturer, is not guaranteed or endorsed by the publisher.

## Supplementary material

Supplementary Figure 1 Fatty acid metabolism, chronic inflammation, and hypoxia promote the occurrence and development of tumor.

Supplementary Figure 2
**(A, B)** GO and KEGG enrichment analysis of integration DEGs. **(A)** GO enrichment based of integration DEGs. **(B)** KEGG enrichment based of integration DEGs.

Supplementary Figure 3
**(A–F)** Prognostic analysis of the risk score. **(A–F)** Risk heatmap of 15 genes, risk score, and survival status in the train group and external validation group.

Supplementary Figure 4
**(A–C)** Validation of a nomogram. **(A)** Calibration curves of the nomogram for external validation group. **(B, C)** Univariate and multivariate Cox regression analysis of nomogram for predicting overall survival in the external validation group.

Supplementary Figure 5 The correlation between the expression of 10 hub genes and the prognosis of bladder cancer.

Supplementary Figure 6 FASN, GALK1, MECR, and MYC immunohistochemistry verification between tumor and normal tissues.
