# Supplementary figures and images for: The prognosis of bladder cancer is affected by fatty acid metabolism, inflammation, and hypoxia
Source: Front Oncol. 2022 Nov 21;12:916850. doi: 10.3389/fonc.2022.916850 (PMC9720300; doi:10.3389/fonc.2022.916850)

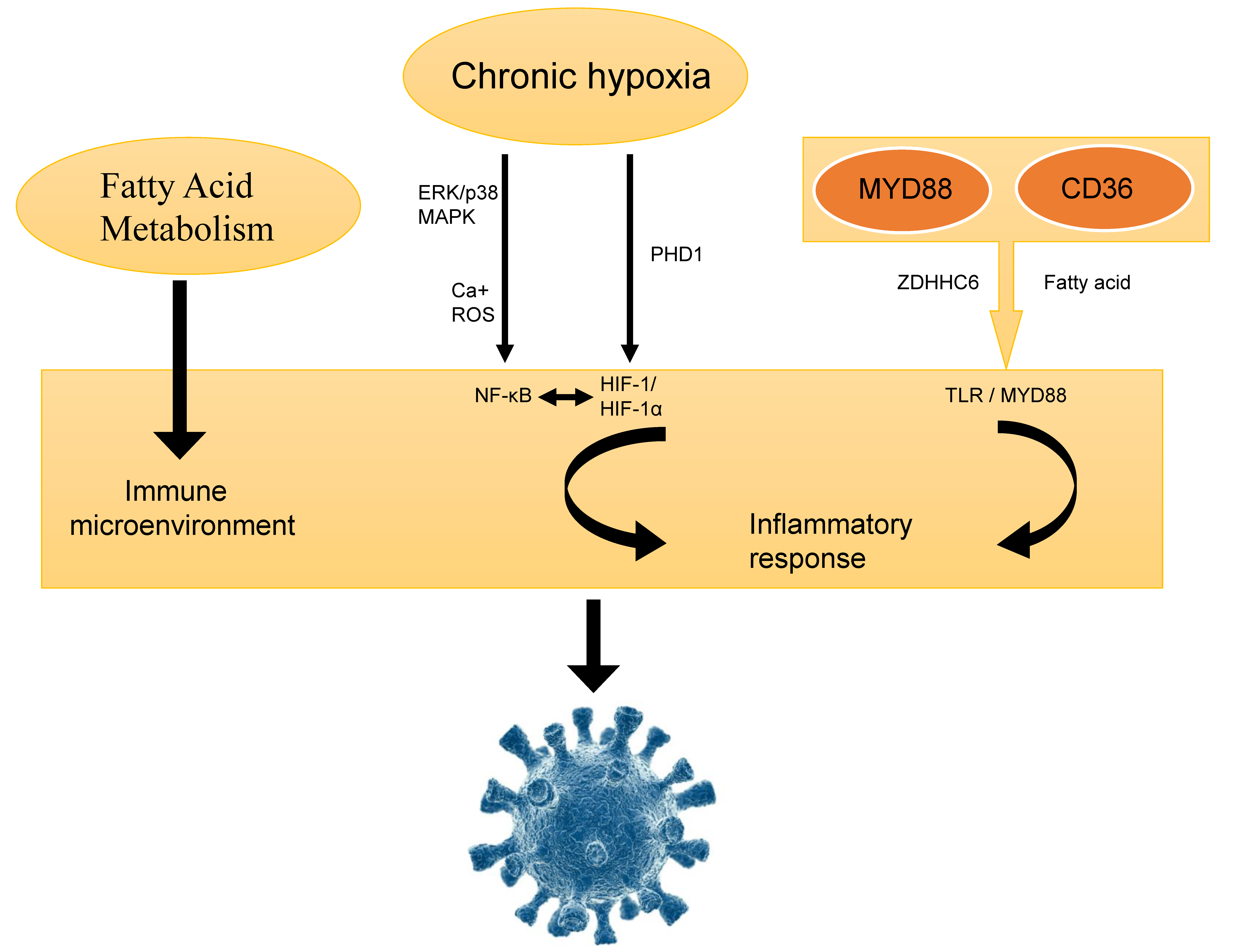

Supplement: Supplementary Figure 1 — Fatty acid metabolism, chronic inflammation, and hypoxia promote the occurrence and development of tumor. [file Image_1.tif]

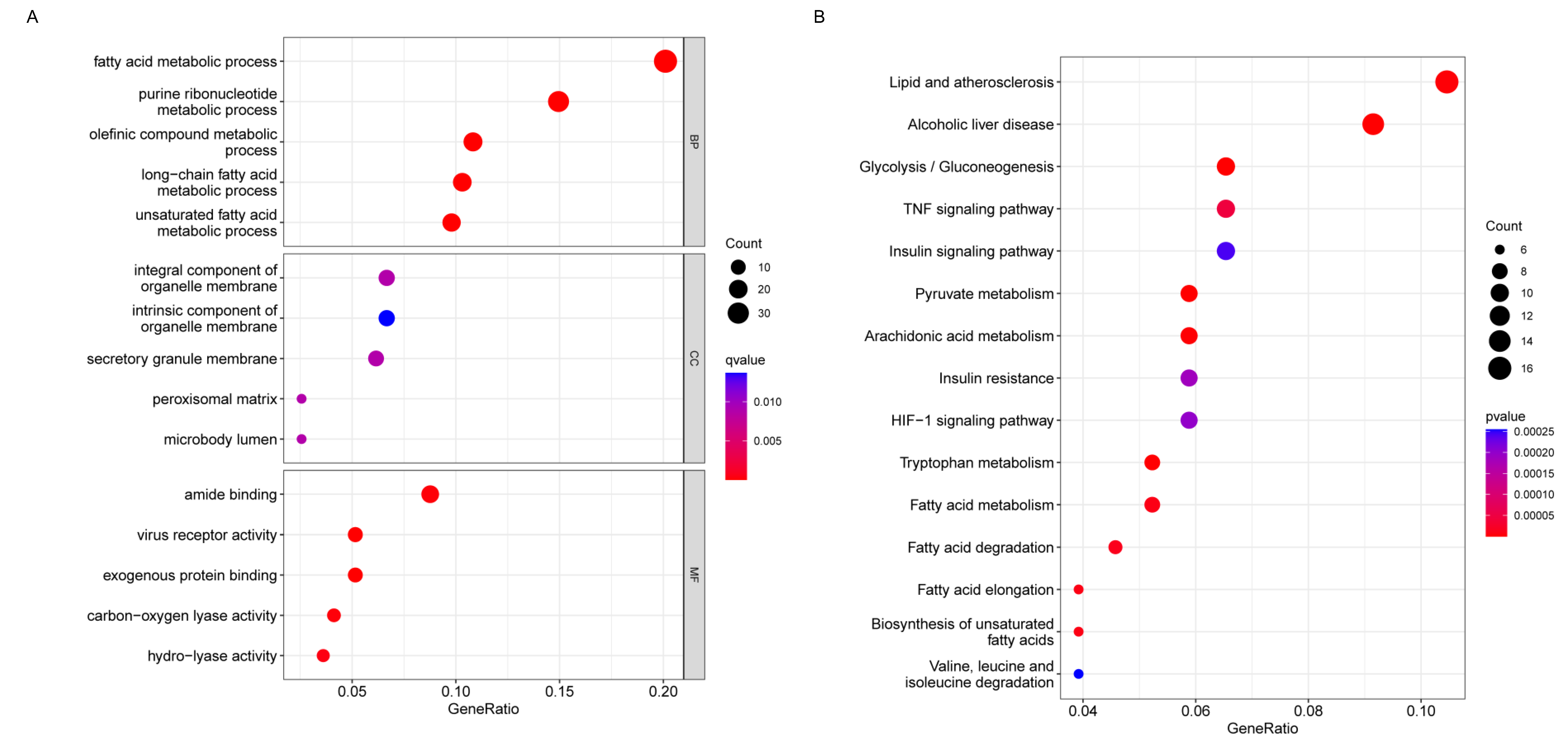

Supplement: Supplementary Figure 2 — (A, B) GO and KEGG enrichment analysis of integration DEGs. (A) GO enrichment based of integration DEGs. (B) KEGG enrichment based of integration DEGs. [file Image_2.tif]

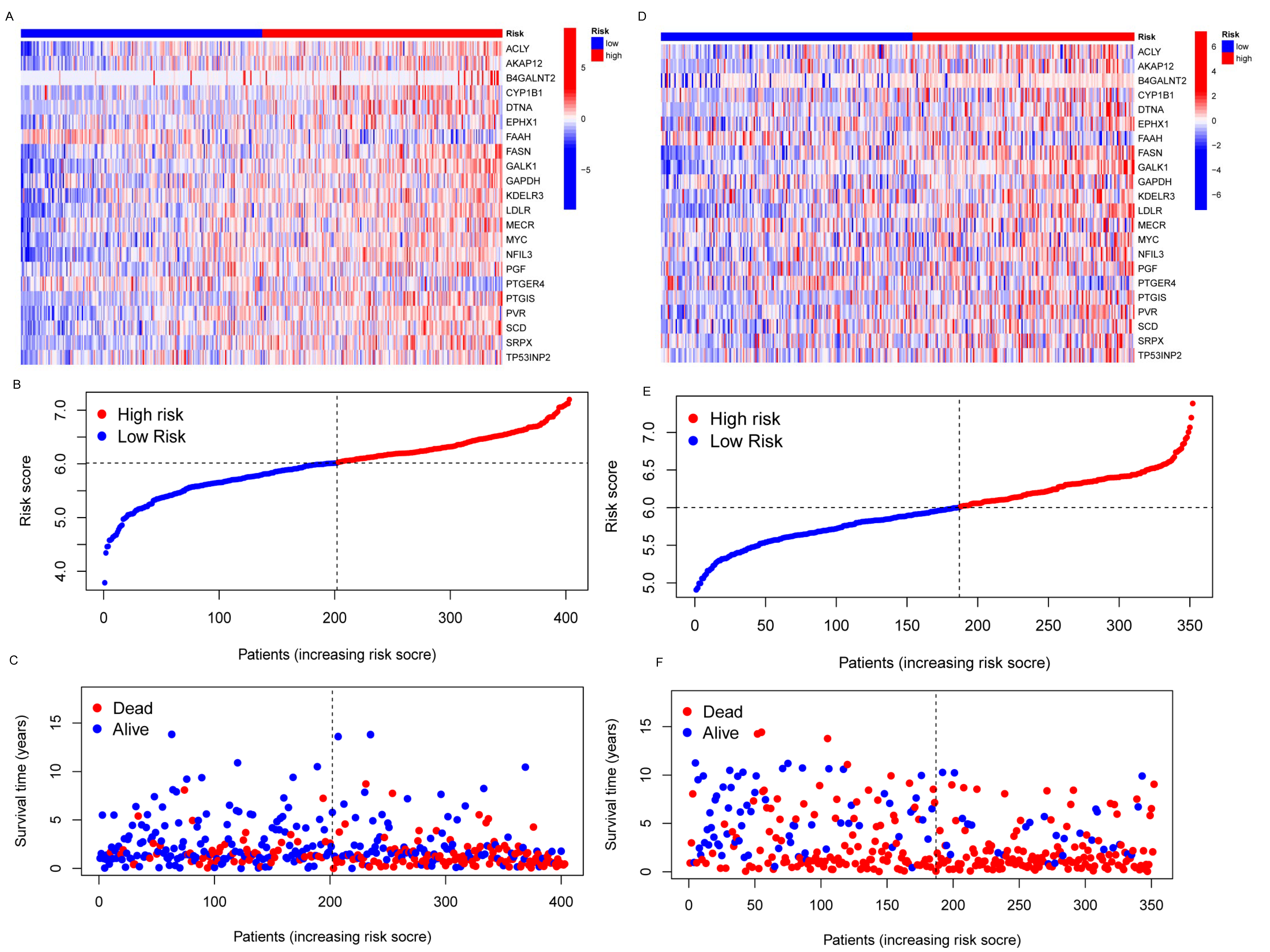

Supplement: Supplementary Figure 3 — (A–F) Prognostic analysis of the risk score. (A–F) Risk heatmap of 15 genes, risk score, and survival status in the train group and external validation group. [file Image_3.tif]

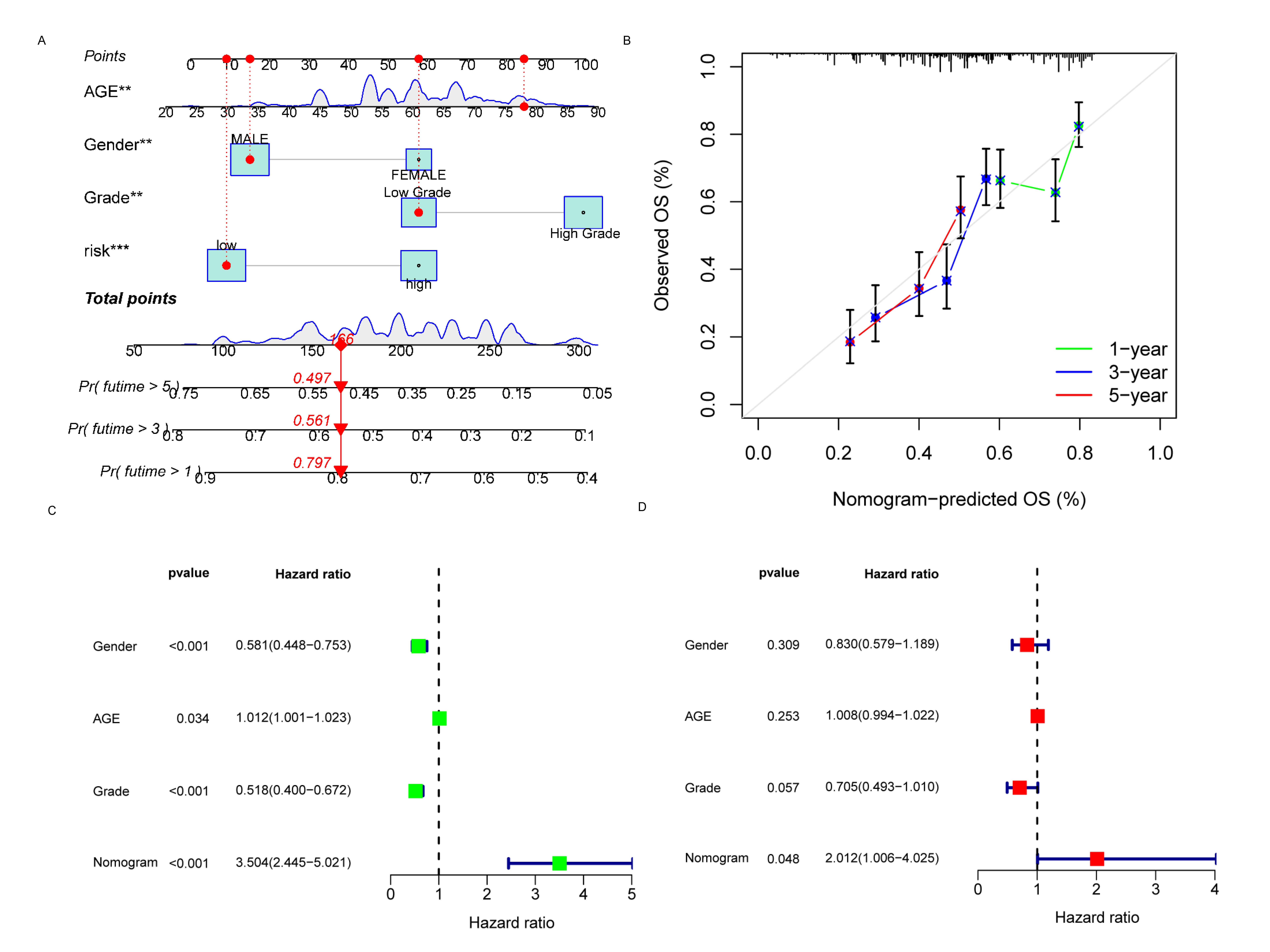

Supplement: Supplementary Figure 4 — (A–C) Validation of a nomogram. (A) Calibration curves of the nomogram for external validation group. (B, C) Univariate and multivariate Cox regression analysis of nomogram for predicting overall survival in the external validation group. [file Image_4.tif]

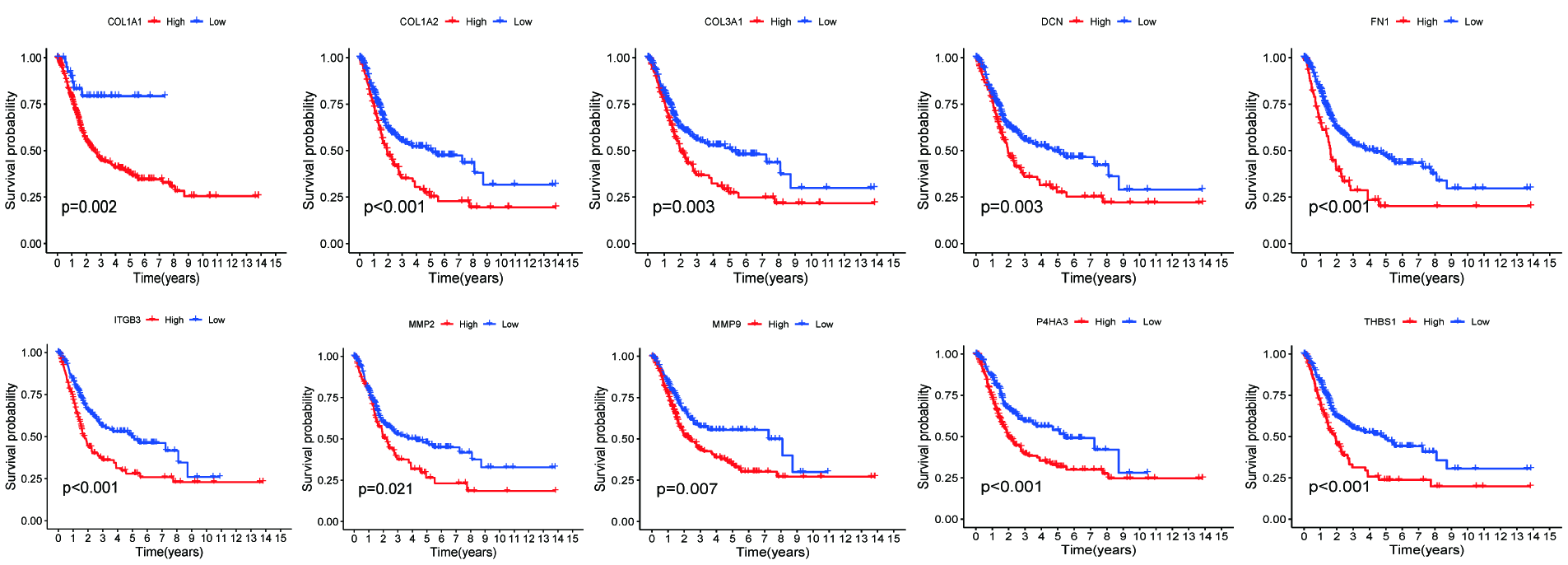

Supplement: Supplementary Figure 5 — The correlation between the expression of 10 hub genes and the prognosis of bladder cancer. [file Image_5.tif]

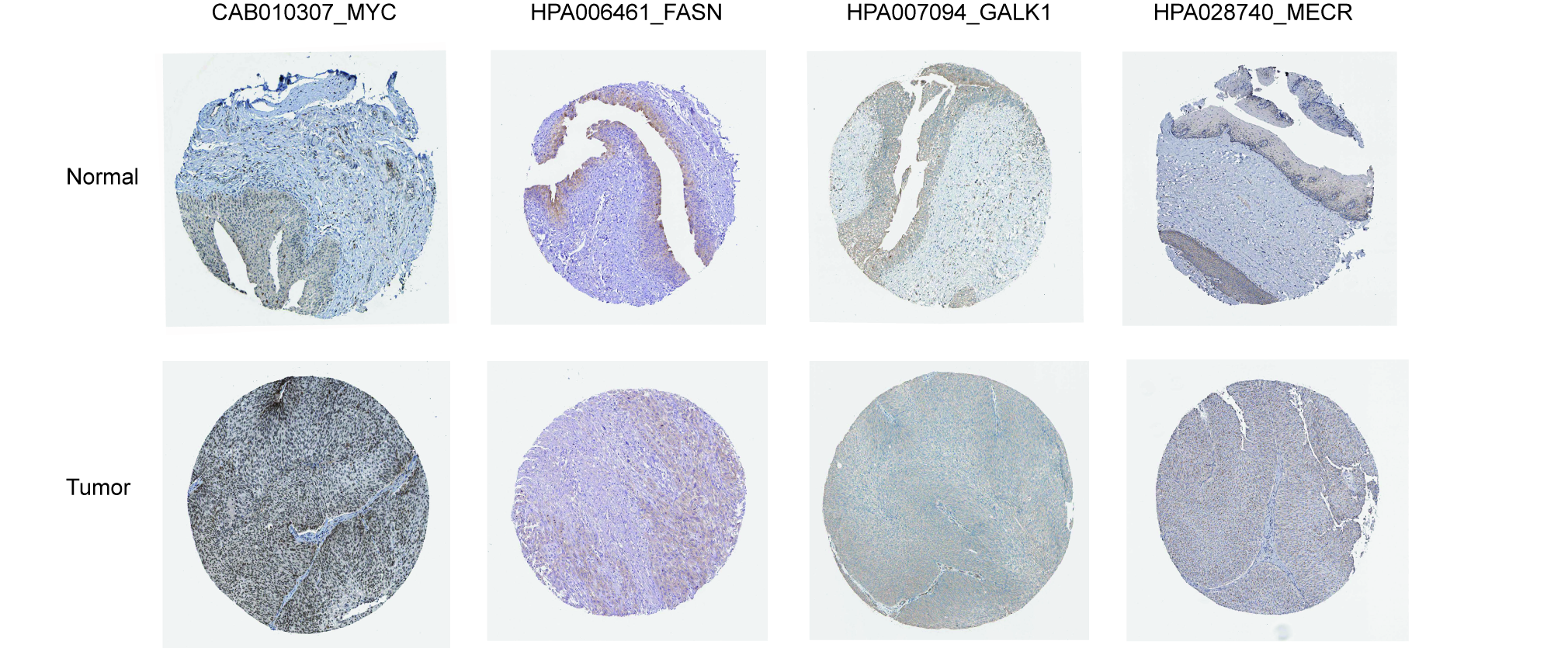

Supplement: Supplementary Figure 6 — FASN, GALK1, MECR, and MYC immunohistochemistry verification between tumor and normal tissues. [file Image_6.tif]
