# Supplementary material for: The prognosis of bladder cancer is affected by fatty acid metabolism, inflammation, and hypoxia
Source: Front Oncol. 2022 Nov 21;12:916850. doi: 10.3389/fonc.2022.916850 (PMC9720300; doi:10.3389/fonc.2022.916850)
Supplement: Supplementary file 7 [file DataSheet_1.docx]

**TABLE. S1 Clinical Information.**

| Covariates | Type | Number=757 |
| --- | --- | --- |
| Age | <=60 | 153 (20.2%) |
| Age | >60 | 417 (55.1%) |
| Age | unknow | 187 (24.7%) |
| Gender | female | 134 (17.7%) |
| Gender | male | 436 (57.6%) |
| Gender | unknow | 187 (24.7%) |
| Grade | High Grade | 529 (69.9%) |
| Grade | Low Grade | 131 (17.3%) |
| Grade | unknow | 97 (12.8%) |
| T | T0 | 1 (0.1%) |
| T | T1 | 93 (12.3%) |
| T | T2 | 166 (21.9%) |
| T | T3 | 254 (33.6%) |
| T | T4 | 88 (11.6%) |
| T | unknow | 155.(20.5%) |
| M | M0 | 351 (46.4%) |
| M | M1 | 18 (2.4%) |
| M | unknow | 388 (51.2%) |
| N | N0 | 384 (50.7%) |
| N | N1 | 53 (7.0%) |
| N | N2 | 82 (10.8%) |
| N | N3 | 9 (1.2%) |
| N | unknow | 229 (30.3%) |

**TABLE. S2 Risk Score.**

| Gene | Coef |
| --- | --- |
| ACLY | 0.0306172267107564 |
| AKAP12 | 0.0332751679647625 |
| B4GALNT2 | 0.183173628349571 |
| CYP1B1 | 0.0343599140153431 |
| DTNA | 0.0476110468819455 |
| EPHX1 | 0.0561497045030001 |
| FAAH | -0.0484374013741379 |
| FASN | 0.182433951300823 |
| GALK1 | 0.273629391302127 |
| GAPDH | 0.0240818060862691 |
| KDELR3 | 0.0257148111482575 |
| LDLR | 0.0689508132233509 |
| MECR | 0.130889882688542 |
| MYC | 0.0614058684612857 |
| NFIL3 | -0.0283475792719741 |
| PGF | 0.0454621824630672 |
| PTGER4 | -0.0932172917443264 |
| PTGIS | 0.0511024533174772 |
| PVR | 0.028289265755746 |
| SCD | 0.0495006781965414 |
| SRPX | 0.0197259066556705 |
| TP53INP2 | 0.0172263783055672 |
